# Homologous Recombination Deficiency Score Determined by Genomic Instability in a Romanian Cohort

**DOI:** 10.3390/diagnostics13111896

**Published:** 2023-05-29

**Authors:** Viorica-Elena Rădoi, Mihaela Țurcan, Ovidiu Virgil Maioru, Andra Dan, Laurentiu Camil Bohîlțea, Elena Adriana Dumitrescu, Adelina Silvana Gheorghe, Dana Lucia Stănculeanu, Georgia Thodi, Yannis L. Loukas, Ileana-Delia Săbău

**Affiliations:** 1 Department of Medical Genetics, “Carol Davila” University of Medicine and Pharmacy, 020021 Bucharest, Romania; viorica.radoi@umfcd.ro (V.-E.R.); ovidiu-virgil.maioru@rez.umfcd.ro (O.V.M.); dandra.crgm@gmail.com (A.D.); laurentiu.bohiltea@umfcd.ro (L.C.B.); ileana-delia.sabau@drd.umfcd.ro (I.-D.S.); 2 “Alessandrescu-Rusescu” National Institute for Maternal and Child Health, 20382 Bucharest, Romania; 3 Personal Genetics, 010987 Bucharest, Romania; 4 Sanador, 011026 Bucharest, Romania; 5 Department of Oncology, “Carol Davila” University of Medicine and Pharmacy, 020021 Bucharest, Romania; elena-adriana.dumitrescu@drd.umfcd.ro (E.A.D.); dana.stanculeanu@umfcd.ro (D.L.S.); 6 Department of Medical Oncology I, Institute of Oncology “Prof. Dr. Al. Trestioreanu” Bucharest, 022328 Bucharest, Romania; 7 Neoscreen Diagnostic Laboratory, Voreiou Ipeirou, 15235 Athens, Greece; lab@neoscreengr.com; 8 School of Pharmacy, University of Athens, Panepistimiolopis, 15771 Zografou, Greece; yannis@neoscreengr.com

**Keywords:** homologous recombination deficiency, Romanian cohort, ovarian cancer

## Abstract

The Homologous Recombination Deficiency (HRD) Score, determined by evaluating genomic instability through the assessment of loss of heterozygosity (LOH), telomeric allelic imbalance (TAI), and large-scale state transitions (LST), serves as a crucial biomarker for identifying patients who might benefit from targeted therapies, such as PARP inhibitors (PARPi). This study aimed to investigate the efficacy of HRD testing in high-grade serous ovarian carcinoma, tubal, and peritoneal cancer patients who are negative for somatic BRCA1 and BRCA2 mutations and to evaluate the impact of HRD status on Bevacizumab and PARPi therapy response. A cohort of 100 Romanian female patients, aged 42–77, was initially selected. Among them, 30 patients had unsuitable samples for HRD testing due to insufficient tumor content or DNA integrity. Using the OncoScan C.N.V. platform, HRD testing was successfully performed on the remaining 70 patients, with 20 testing negative and 50 testing positive for HRD. Among the HRD-positive patients, 35 were eligible for and benefited from PARPi maintenance therapy, resulting in a median progression-free survival (PFS) increase from 4 months to 8.2 months. Our findings support the importance of HRD testing in ovarian cancer patients, demonstrating the potential therapeutic advantage of PARPi therapy in HRD-positive patients without somatic BRCA1/2 mutations.

## 1. Introduction

In both external and internal environments (endogenous, free oxygen radicals), numerous physical, chemical, and biological mutagenic factors can cause specific lesions at the DNA sequence level. These factors can impact the expression and function of cellular proteins by causing genomic instability [1].

Changes in the genetic material can also influence the correct replication of the cellular genome.

A cell can experience 10,000–1,000,000 lesions in its DNA sequence per day. Endogenous cellular processes generate oxygen free radicals that affect genetic material 10,000 times per day.

The cell’s repair mechanisms respond and correct the changes most of the time, but pathogenic variations that emerge at the level of oncogenes, tumor suppressor genes, and genes that control the cell cycle have an impact on cell growth and proliferation, potentially resulting in cancer [2].

After breast cancer, ovarian cancer is the second most common and most fatal location of neoplasms of the female genital tract. Surgical therapy, chemotherapy using taxanes and platinum-based drugs, targeted therapy, hormone therapy, and radiation therapy are examples of traditional treatment plans [3]. Surgery, which aims to remove as much of the tumor as possible (a process known as debulking), continues to be a staple of ovarian cancer treatment. Optimal debulking procedures are linked to higher survival rates. The ovaries, fallopian tubes, uterus, surrounding lymph nodes, and the omentum may all need to be removed during surgery, depending on the severity and spread of the disease. Neoadjuvant chemotherapy and major debulking surgery have comparable efficacy when used to the utmost degree, but their toxicity profiles are different.

Hyperthermic intraperitoneal chemotherapy (HIPEC) combined with interval cytoreductive surgery improves the outcomes in individuals receiving neoadjuvant chemotherapy for stage III epithelial ovarian cancer. 

Additionally, there are treatments that target specific cancer cell growth- and division-promoting factors. For instance, PARP inhibitors such as olaparib, niraparib, and rucaparib have demonstrated efficacy in treating some kinds of ovarian cancer, notably those with BRCA mutations, with effectiveness in improving progression-free survival. In some instances of recurrent ovarian cancer, hormone treatments such as letrozole or tamoxifen may be administered, notwithstanding their rarity. Additionally, treatment with immune checkpoint inhibitors such as pembrolizumab has produced encouraging preliminary results. Radiation therapy may occasionally be utilized in specific circumstances, despite the fact that it is not frequently employed due to the disease’s widespread distribution and the efficacy of other treatments.

Diagnostic, prognostic, predictive, and responsive types of biomarkers are divided into categories because they are essential for early detection and higher survival rates. However, the low sensitivity and specificity of many biomarkers present difficulties. At present, the three most frequently utilized biomarkers are mesothelin, human epididymis protein 4 (HE4), and CA125. In order to investigate the potential of these and other biomarkers (OVA1, DOvEEgene) in OC identification and management, numerous studies and clinical trials are being carried out globally.

According to the most recent genetic studies, BRCA1/2 germline mutations are the most potent genetic risk factors for epithelial ovarian malignancies and are present in 6–15% of women who have been diagnosed with the disease. As BRCA1/2 carriers with epithelial ovarian cancer respond better to platinum-based chemotherapies than non-carriers, the BRCA1/2 status can be used to inform patients about their expected survival. Even though the disease is typically diagnosed at a later stage and with a higher grade, this results in a higher survival rate.

Malignant ovarian lesions can be either primary (which develop from healthy ovarian tissues) or secondary (which spread from another main cancer). Ovarian stromal tumors, which may include germ cell tumors, sex cord stromal tumors, ovarian carcinosarcomas, and other less common kinds, are primary lesions. Epithelial ovarian carcinoma accounts for 70–80% of all ovarian malignancies. Metastases in the ovaries are fairly prevalent, and endometrial, breast, colon, stomach, and cervix tumors are common causes [4].

There are various subtypes of epithelial ovarian cancer, with the serous subtype being the most prevalent form. Eight out of ten (80%) or more cases of ovarian cancer are serious, high-grade, and quickly progressing malignancies. Other, much less frequent, kinds include clear cell, endometrioid, and mucinous ovarian cancers. These are less aggressive and are typically detected earlier than serous cancers [5].

Endometriosis or borderline tumors with low malignant potential are examples of indolent and genetically stable tumors that are regarded as the primary precursor lesions for type I epithelial ovarian cancers, which are usually caused by these lesions. Type II epithelial ovarian cancers, on the other hand, are regarded as being physiologically aggressive tumors from the beginning and have a predisposition to metastasis from small-volume initial lesions. High-grade serous, which develops via the type II pathway, contains p53 and BRCA mutations.

Two basic explanations have been presented to explain the development of resistance to PARPi therapy. The first one entails the acquisition of additional alterations that restore HRR at the level of cancer cells, either by re-expressing a gene that has been transcriptionally inactivated by mutations or epigenetics, or by (rewiring) the emergence of alternative responses to DNA damage [6,7]. The development of other cellular and nuclear processes, such as the reduction in PARP capture, the protection of the replication fork, and the increase in drug efflux (through mutations in the ABCB1 gene, which codes for the pump of multi-drug efflux MDR1, mutations leading to increased ABCB1 expression) are what determine the second group of resistance mechanisms, rather than the restoration of HRR [8,9,10].

By preventing PARP proteins from being modified, PARP inhibitors stop PARP from dissociating from DNA single-strand breaks. More single-strand breaks build up as a result of the attachment of more repair proteins being blocked. These single-strand breaks become double-strand breaks in reproducing cells. The PARP1 and PARP2 enzymes are bound to damaged DNA by the PARP inhibitors, which also prevent single-strand repair. This “parp capture” has a significant cytotoxic effect. The replication fork is stopped by PARP-DNA complexes, which also cause an accumulation of further DNA double-strand breaks. The homologous recombination repair (HRR) process fixes these double-strand breaks in healthy cells [11,12,13].

Synthetic lethality is the idea of combining two conditions to compel cell death, and Ashworth first documented its application in cancers with impaired DNA repair or changed checkpoint regulation in 2008 [14].

The role of PARP in the immune system has become increasingly apparent. One of the ways PARP contributes to immune function is through its involvement in the inflammatory response. PARP1 is activated by DNA damage and oxidative stress, both of which are common during inflammation. Once activated, PARP1 helps regulate the expression of various inflammatory genes.

PARP also appears to play a role in the adaptive immune response. Some studies suggest that PARP1 is involved in the process of class switch recombination, a mechanism the immune system uses to enhance its response to infection or vaccination. Additionally, PARP is believed to play a role in the maturation of T cells, a key component of the adaptive immune response.

Given the role of PARP in DNA repair, it’s not surprising that PARP deficiency or inhibition can have significant effects. In normal cells, PARP inhibition does not usually cause problems because these cells have other DNA repair mechanisms they can rely on. However, cancer cells, particularly those with mutations in certain DNA repair genes, such as BRCA1 or BRCA2, are more dependent on PARP (synthetic lethality).

Due to PARP’s function in the immune system, immunomodulatory effects of its blockage may also be possible. As PARP1 contributes to inflammation, for instance, its suppression may have anti-inflammatory effects. According to certain preclinical research, PARP inhibitors can improve symptoms in inflammatory disease model organisms by lowering the production of pro-inflammatory cytokines and chemokines.

PARP inhibition has complicated and poorly understood effects on the immune system. While certain effects, such as decreased inflammation, may be advantageous, others may be harmful. For instance, PARP inhibition may reduce the immunological response if it hinders the development of T cells. 

Epigenetic modifications may potentially be responsible for the homologous repair system’s malfunctioning operation. Approximately 11% of ovarian cancer cases have hypermethylation at the BRCA1 gene’s promoter level, whereas only 3% of cases have it at the RAD51C gene level [15,16]. Genes’ promoter CpG islands become hypermethylated, which hinders transcription and lowers gene expression. The contrasting results from different research make it difficult to determine a relationship between the hypermethylation of the BRCA1 or RAD51C genes and how well they respond to treatment with platinum salts or when PARP is active [17,18].

The information from the entire genome, including chromosomal abnormalities and mutations brought on by genomic instability caused by HRD, is captured by “genomic scars” and multiple gene sequencing assays. Thus, a wider patient population could now use PARPi thanks to the examination of these intricate genetic modifications [14,19].

When Olaparib and Bevacizumab are used as the first-line maintenance therapy for ovarian cancer, HRD testing is used. These tests are helpful in the first stages of treatment even if they detect prior events rather than necessarily reflecting the DHR status of the tumor at that time. However, if an H.R. restoration event occurred during tumor progression under the strain of numerous lines of chemotherapy, they can result in an incorrect judgment regarding the efficacy of the treatment. The development of tests that accurately reflect the HRD tumor phenotype in real time is therefore imperative [12,14,20,21].

Since no single test is ideal, each of these tests contribute to understanding the HRD phenotype of a tumor. Given that the results set must be compatible with the time of treatment initiation, a combination of several of these tests may have a superior predictive value and should be elaborated upon [16].

At the level of the human species, there are a number of DNA damage repair systems that function at the level of intracellular signaling transduction, transcriptional regulation, cell cycle control points, apoptosis induction, and more [22].

These repair mechanisms are classified into two large categories:

A. A direct, enzymatic, rapidly exhaustible repair that acts specifically to correct the modifications brought on by various alkylating agents.
B. Multiple subtypes of excision repair:
  - Repair by excision of a single nitrogenous base (base excision repair, BER) for chain break or small change changes (non-bulky, small);
  - Repair by excision of a nucleotide fragment (20–30 nucleotides) for larger DNA lesions that cause double helix distortion;
  - Reconstruction of lesions caused by mismatch repair and small insertions/deletions [23].

DNA strand breaks can be repaired through homologous recombination repair (HRR) or (in extreme cases) non-homologous end joining (NHEJ).

DNA double-strand breaks will be treated by alternate, but error-prone, repair mechanisms, such as the NHEJ when HRR function is absent, as in BRCA-mutant cells. This results in an accumulation of genomic instability and, eventually, cancer cell death. NHEJ primarily takes place in the G1 phase and is faster than HRR. However, recent research has shown that NHEJ continues to function all the way through the cell cycle. Beyond the well-known proteins, such as Ku70/80, DNA-PKcs, Artemis, DNA pol /, DNA ligase IV-XRCC4, and XLF, new proteins, such as PAXX, MRI/CYREN, TARDBP of TDP-43, IFFO1, ERCC6L2, and RNase H2, are also involved in the NHEJ. Among these, MRI/CYREN plays a dual role in the cell cycle, stimulating NHEJ in the G1 phase while inhibiting the pathway in the S and G2 phases.

The proteins involved in the DNA damage repair systems are encoded by genes that detect and correct chain breaks and chromosomal by activating multiple cellular signaling pathways [24].

When a mutation at the genetic level first occurs, the cell cycle is stopped (preventing the cell from producing additional abnormal daughter cells), and only then is an attempt made to repair the specific damage. If this is successful and the mechanism of programmed cell death (apoptosis) is not activated, the cell resumes its cell cycle.

The repair systems in cancerous cells are impaired, resulting in genomic instability and tumorigenesis through the accumulation of additional mutations at the vulnerable DNA level [7,25,26].

Targeting the specific proteins involved in repair in neoplastic cells that have deficiencies at this level represents a promising strategy in personalized cancer therapy.

Defects occurring at the level of genes that code for the proteins involved in repair systems can occur at several levels:

A. Abnormalities in the repair of strand breaks and replication errors—the most frequently involved are mutations in the BRCA1 and BRCA2 genes that act through the homologous repair mechanism, forming together with other proteins (encoded by the PALB2, BRIP1, RAD51C, RAD51D genes) the complex BASC (BRCA-associated protein) involved in the recognition and repair of abnormal DNA structures. Pathogenic variants in the BRCA1 and BRCA2 genes appear in different types of cancer, among which they are more frequently found in breast, ovarian, pancreatic, or prostate cancer [27].
B. Defects in the genes that control the cell cycle and identify lesions in the genetic material (ATM, ATR, CHEK1, and CHEK2).
C. Pathogenic variants in the genes of the MMR system (MSH2, MSH3, MSH6, MLH1, PMS2) that lead to an increase in the mutational load (burden) in tumor cells [11,28].

### HRD Testing

HRD genetic testing (including loss of heterozygosity—LOH, large scale state transitions—LST and telomeric allelic imbalance—TAI) has prognostic value (progression-free survival and overall survival) and an impact on the comprehensive treatment plan, which has been validated by a large number of clinical studies [29,30,31]. 

Tumor characterization by HRD score testing identifies possible gene mutations (at the level of the BRCA1 and BRCA2 genes in the tumor tissue) and reveals genomic instability through LOH, LST, and TAI (collateral damage).

LOH—loss of heterozygosity;

TAI—regions at the level of chromosomal telomeres with allelic imbalance, i.e., the two alleles of a gene are expressed at different levels in the cell;

LST—genomic changes that involve chromosomal tears/breaks.

Currently, BRCA1 and 2 mutation status testing is recommended for patients confirmed with ovarian, tubal, or peritoneal cancer.

The benefits and clinical implications of HRD testing include:

- Predispositional insights: HRD testing can identify a person’s family members’ risk of getting ovarian cancer by detecting germline mutations of the BRCA1/2 genes. It can also help identify ovarian cancer patients at risk of other cancers;
- Prognostic insights: The test provides insight into the course of the disease and identifies whether the tumor has variants that may cause HRD;
- Treatment insights: Testing helps guide and plan a comprehensive treatment plan and determine whether targeted treatment, such as PARP inhibitors, will benefit the patient [32,33].

The HRD Score is a molecular analysis approach that quantifies the cellular rate of acquisition of chromosome breaks using specific quantitative models called “genomic scars”.

Cells with HIGH HRD values have been shown to be more sensitive to poly (ADP-ribose) polymerase (PARPi) inhibitors and platinum therapy [34].

The use of small therapeutic molecules that inhibit the activity of PARP to repair DNA lesions in tumor cells has been approved since 2014. There are currently four molecules approved by the F.D.A. (olaparib, niraparib, rucaparib, talazoparib) [13,21,35,36].

HRD testing allows the identification of approximately 50% of patients eligible for treatment with PARPi and monoclonal antibodies [37].

Studies for testing HRD as a biomarker for response to PARPi (PAOLA, PRIMA) showed that patients with BRCA1/2 mutations or mutations in other genes involved in HRR had a better response to treatment than patients without mutations [38,39].

Due to the strong predictive and prognostic value, the European Expert Consensus recommends BRCA and HRD testing for patients diagnosed with advanced ovarian cancer [32].

The use of PARPi in patients with E.O.C. is increasing, particularly as clinical trial approvals move to the frontline. Despite the unprecedented benefits seen in some groups, not all patients benefit, and treatment failure is common due to de novo or acquired resistance. As the population of PARP-resistant patients increases, there is an urgent need to better understand and clinically validate the proposed mechanisms of acquired resistance [40,41,42].

## 2. Materials and Methods

Testing was conducted using the determination of the Genomic instability score, which was determined by adding the LOH, TAI and LST scores using the OncoScan C.N.V. platform (Thermo Fisher Scientific, Waltham, MA, USA).

Genetic material was extracted using the QIAamp DNA FFPE Tissue Kit (Qiagen, Hilden, Germany). The concentration was determined using the Qubit TM dsDNA H.S. Assay Kit (Thermo Fisher Scientific, Waltham, MA, USA) on a Qubit fluorometer. The extracted genetic material was tested on OncoScan C.N.V. on an Affymetrix platform (Thermo Fisher Scientific, Waltham, MA, USA) following the manufacturer’s instructions.

SNP Arrays are commonly used HRD-score methodologies designed to detect C.N.V.s and LOHs, and such is the Affymetrix’s Oncoscan platform. This kit is based on MID Detector (Molecular Inversion Probe) technology and has the ability to detect “genomic scars” in degraded DNA, such as that of FFPE cancer tissue. The results are analyzed using the Chromosome Analysis Suite (ChAS) program with the GRCh37 reference genome [43].

A HRD-positive result is due to somatic mutations in the BRCA1 and BRCA2 genes and/or Genomic instability score ≥ 42. A HRD-negative sample is defined by both wildtype somatic BRCA genes and a Genomic instability score < 42 [44]. The patients who already had somatic mutations in BRCA genes could be good candidates for Bevacizumab and Parpi therapy; therefore, due to limited funds, we selected only the wildtype somatic BRCA1 and BRCA2 patients for our study [45,46].

Archival formalin-fixed paraffin-embedded (FFPE) tissue from patients with primary diagnosis of ovarian cancer outside of our department was requested from external pathology departments whenever the quality and/or quantity of tumor tissue was not sufficient for HRD testing.

The selection of patients was made using the recommendations of the ESMO guidelines [18], and we included patients confirmed with ovarian, tubal, or peritoneal cancer. In addition, 23 of the patients already had metastases. One important inclusion criterion was that the patients had to be BRCA1/2 negative at a prior testing from tumor cells.

For molecular-tissue-based HRD tests, representative tumor area selection and assessment of the percentage of malignant cells, necrosis, and inflammatory component is of fundamental importance. Typically, a minimum of 30% tumor component is recommended to guarantee the detection of a variant through molecular techniques [46].

## 3. Results

We present our Romanian cohort of 100 female patients of Caucasian descent, aged 42–77, who were diagnosed with high-grade serous ovarian carcinoma, tubal, or peritoneal cancer. The patients selected tested negative for somatic BRCA1 and BRCA2 and were receiving therapy with first-line chemotherapy and Bevacizumab.

**Figure 1:**
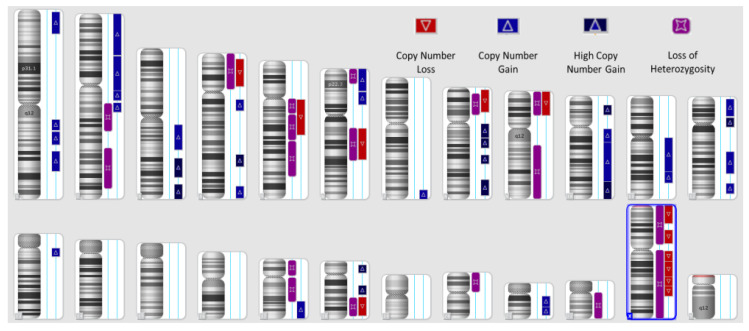
Schematic representation of CNVs (loss and gain events) and LOH in a high-HRD sample.

**Figure 2:**
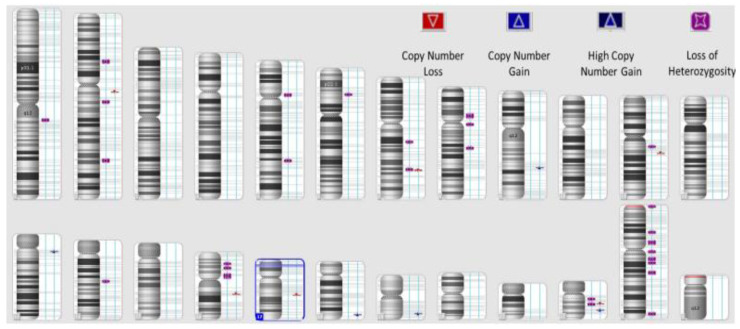
Schematic representation of CNVs (loss and gain events) and LOH in a HRD-negative sample (HRD = 0).

The initial cohort was made up of 100 patients, from which 30 patients had samples that did not meet the criteria to perform HRD testing. One of the most important criteria is for the FFPE sections to have a minimum tumoral content of 30%, and the samples were analyzed and prepared by the pathology laboratory. Another important criterion is DNA tumoral integrity; the laboratory tested the concentration of extracted DNA in every sample, and there were five samples that tested under 162.4 ng/ul (Qubit measurement). A total of 30% of the samples were thereby inappropriate and unacceptable for further testing. Figure 1 represents a sample example of the schematic representation of CNVs (loss and gain events) and LOH in a high-HRD sample, while Figure 2 presents a HRD-negative sample (Figure 1 and Figure 2).

**Figure 3:**
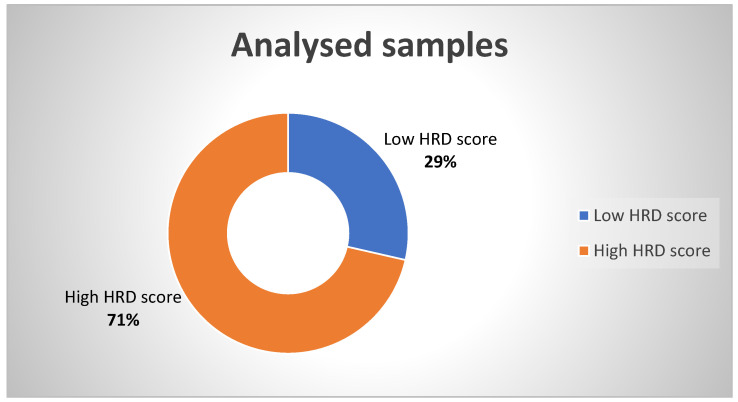
Percentages of High and Low HRD Scores.

We present a report of 70 female patients for whom we performed HRD testing with the following results: 20 patients tested negative and 50 patients tested positive, with a high HRD score (Figure 3).

The patients who had a high HRD score were followed-up, and we present their management and overall outcome.

**Figure 4:**
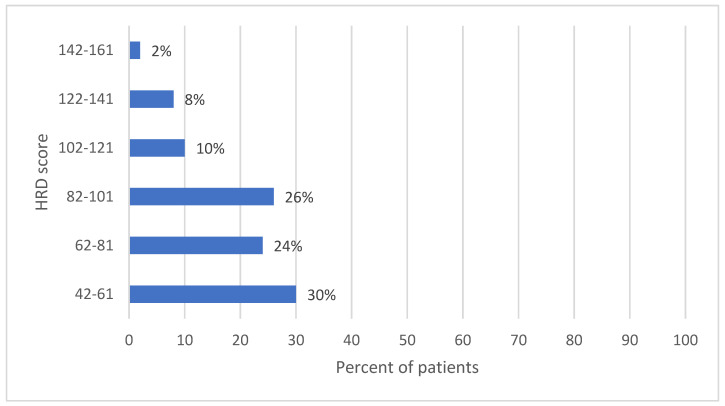
The percent of patients corresponding to each HRD range score.

Most of the patients (15, 30%) had a HRD score between 42 and 61. Overall, 13 patients (26%) were HRD-positive, with a score between 82 and 101, while 12 patients (24%) had a score between 62 and 81. The highest HRD scores (142–161) were present in only 2% of the analyzed cases (1 patient) (Figure 4).

**Figure 5:**
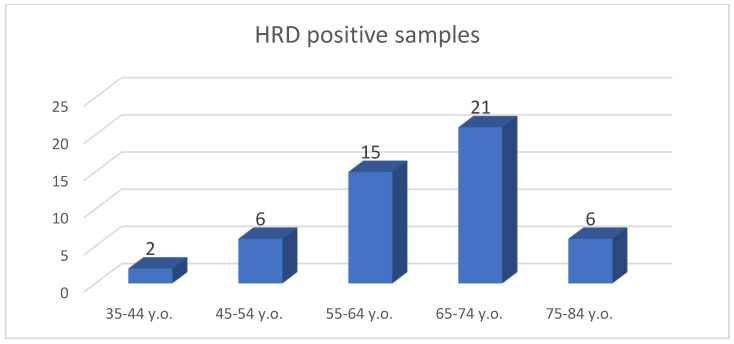
Schematic representation of total HRD-positive samples based on age intervals of patients.

The age range was 42–77 years old; thus, the mean age was 63.16 years old (Figure 5).

The most elevated score was LST for every patient, but all of the scores together reported the genomic scars of the tumoral tissue in a more accurate manner than when taken separately.

Most of the patients were diagnosed as stage FIGO IIIC ovarian cancer (29, 58%; 95% CI: 43.21%–71.81%), having the highest median HRD score (85, Std. Dev. = 25.42) (Table 1).

Patients who were eligible for PARPi maintenance therapy had to have a complete response (CR) or a partial response (PR) from platinum chemotherapy. From 50 patients, 35 benefited from PARPi therapies, with overall, median progression-free survival (PFS) was elevated from 4 months to 8.2 months (HR 0.38 94% CI 0.35–0.47; *p* < 0.001).

## 4. Discussion

The present study showed that the implementation of HRD testing is feasible, despite the fact that it is currently conducted on pharma vouchers, and the overall turnaround time for receiving the HRD results was acceptably long for the treatment decision. However, the most critical issue for performing HRD testing is the amount of tumor tissue that needs to be available. A considerably large number of patients did not receive a sufficient HRD test result due to a lack of available tumor tissue, and some of these patients may not have received optimal treatment because of this.

BRCA1/2 mutant malignancies have a better prognosis than BRCA1/2 wildtype tumors, which has long been acknowledged. Even when PARPi is not used, this still applies to HRD. HRD testing is important for prescribing Bevacizumab and PARPi (Olaparib) therapy [12,47,48,49]. Tumors from patients who are eligible for Bevacizumab treatment and who have high-grade carcinomas should undergo HRD testing [47].

At present, patients with early ovarian cancer or those with low-grade carcinomas are not eligible for maintenance therapy with Bevacizumab and Olaparib [39]. In the PRIMA trial, for instance, the median PFS for the HRD/(BRCA1/2 wildtype) and HRD-negative groups in the placebo arms was 10.9, 8.2, and 5.4 months, respectively. In the subgroup analysis of the PRIMA trial, patients with HRD and somatic BRCA1/2 mutations were shown to have a very good response to maintenance therapy with niraparib alone (H.R.: 0.4 (95% CI 0.27, 0.62)), as were patients who had evidence of an HRD but no BRCA1/2 somatic mutation (H.R.: 0.5 (95% CI of 0.31, 0.83)). Using the whole-genome data from large clinical datasets, it was found that HRD was strongly linked to prolonged OS; this association persisted even when individuals with BRCA1/2 were excluded.

Subgroup analyses of the PAOLA-1 trial showed that maintenance therapy with bevacizumab and olaparib resulted in a median PFS improvement of 19.5 months in patients with HRD-positive tumors (including BRCA1/2 somatic mutations) compared to the placebo-controlled group [50,51,52]. Patients with HRD-positive tumors (without BRCA1/2 somatic mutations) had a median PFS improvement of 11.5 months, which was also statistically significant. In the same study, the results showed that patients who were HRD-negative did not show an improvement in their PFS with the addition of olaparib to bevacizumab [50,51,52,53].

On the other hand, HRD testing should begin as soon as feasible to allow for speedier availability of the test results for firm planning of maintenance therapy. After patients underwent primary surgery and then received six cycles of chemotherapy every three weeks (a duration of roughly 126 days), the results of our study were available after an average of 35 days, which was acceptable and in line with previously reported central genomic analyses.

The lack of cost coverage by the national health system means that not all clinicians are aware of the availability of these tests through vouchers to their patients, which is one of the most significant disadvantages to the speedier receipt of HRD results and access to all patients with high-grade ovarian cancer.

The high proportion of non-eligible HRD tests—in the present article, this occurred for 30% of patients—was another significant logistical concern that was emphasized in the analysis. The absence of sufficient tumor material was the sole cause of the non-significant HRD results. As a significant portion of tumors are frequently already in remission by this point [47], the expectation that enough tumor tissue will be found in the sometimes very small biopsy specimen to perform HRD testing is frequently deceiving. In our study, we were unable to locate enough tumor tissue in 30% of patients.

As the effectiveness and approval status of the maintenance therapy that will be administered later may be largely reliant on the HRD status, it is important that the quantity and quality of the tumor tissue used to establish the diagnosis be high enough. A significant factor in shortening the turnaround time for HRD testing is the amount of tumor tissue present at the time of diagnosis. If the initial test was unsuccessful, new FFPE tumor blocks might have been required to redo the procedure, which would have prolonged the wait for the HRD test result [54,55].

The paraffinization process that had been used had largely damaged the FFPE DNA samples. Additional deterrents to the genetic screening process for mutation signatures may be introduced through the paraffinization procedure’s parameters [56].

The relationship between somatic HRD/BRCA testing and panel testing is another thing to think about. Testing for germline panels comprising HRR genes cannot be skipped because HRD results frequently yield inconclusive information.

However, there are significant academic initiatives underway to verify alternative HRD tests to those that are already accessible in order to reduce the costs and improve the performance.

Taking into consideration that most of the patients in our cohort had a favorable outcome and a higher overall rate of survival, HRD testing is efficient and there should be further studies with a greater number of patients in order to have a clearer image of the Romanian HRD picture.

The HRD tumor status is dynamic over time and under the pressure of treatment. The restoration of the HRR repair processes in tumor cells through the mechanisms of resistance to PARPi treatment is not sufficiently revealed by HRD testing, contributing to the clinical discrepancy, or even the absence of treatment response, even in well-selected cases. New technologies in the field of proteomics, such as mass spectrometry and protein array analysis, have advanced the dissection of the underlying molecular signaling events and the proteomic characterization of ovarian cancer. Proteomics analysis of ovarian cancer, as well as their adaptive responses to therapy, can uncover new therapeutic choices, which can reduce the issue of drug resistance and potentially improve patient outcomes.

Therefore, HRD testing must be permanently optimized, taking into account the controversies of the various testing methodologies and the different limits (cut off) of identification, in order to maximize the benefit of the oncological patient [57,58]. Choosing a “gold-standard” for HRD testing is one of the biggest issues. The latest molecular, clinical, functional, and genomic testing have benefits and drawbacks. The genomic scar HRD assays that have been successfully validated to date are valuable in estimating the extent of the benefit of PARPi and can be used to guide therapy decision-making. They fail to address the complicated and dynamic character of the HRD phenotype, which limits their usefulness (especially in the platinum-sensitive recurrent scenario). Therefore, in order to maximize the potential of PARPi in patients with HGOCs, better biomarkers to determine the patient’s current HR status are required, which may call for composite tests.

The spatial and temporal heterogeneity of the tumor may be a factor that contributes to the variability of the response to PARPi, and future studies are needed to evaluate, through multiple biopsies performed in the same patient, HRD scores that may be different. Repeated sequencing of the tumor or liquid biopsy (as a dynamic follow-up) can contribute to a better approach to the presence or absence of reversible mutations. However, even this sequential testing algorithm does not always clearly indicate the presence or absence of independent mechanisms to restore the HRR repair processes that determine treatment resistance [59,60].

## 5. Conclusions

HRD testing in clinical practice detects “genomic scars” as an indirect measure of genomic instability through DNA damage repair deficiency. The tests validated so far evaluate the percentage of LOH determined through sequencing SNPs or using a score calculated by combining three factors: SNP-LOH, telomere allelic imbalance (TAI), and LST.

PARP inhibitors act on them at the level of DNA single-strand breaks, preventing efficient repair, increasing genomic instability, and thus leading to the death of tumor cells.

Studies to test the HRD as a biomarker for the response to PARPi are needed.

## Figures and Tables

**Table 1:** Distribution of patients according to the stage of ovarian cancer at the moment of diagnosis (and HRD testing); LCL = lower confidence limit, UCL = upper confidence limit.

FIGO Stage of Ovarian Cancer	Frequency	Percent	Exact 95% LCL	Exact 95% UCL	Median HRD Score	Std. Dev.
IIIA	6	12.00%	4.53%	24.31%	59	18.5876
IIIB	6	12.00%	4.53%	24.31%	64.5	38.6506
IIIC	29	58.00%	43.21%	71.81%	85	25.4200
IV	9	18.00%	8.58%	31.44%	73	27.6667
TOTAL	50	100.00%				

## Data Availability

Not applicable.

## Abbreviations

ATM	ataxia telangiectasia mutated
BASC	BRCA1-Associated Genome Surveillance Complex
BARD1	BRCA1 Associated RING Domain 1
BER	base excision repair
BRCA1/2	breast cancer gene 1/2
BRIP1	BRCA1 interacting protein 1
FANC	Fanconi anemia complementation
FFPE	formalin-fixed paraffin-embedded
HRD	homologous recombination deficiency
HRR	homologous recombination repair
LOH	loss of heterozygosity
LST	large scale state transitions
MMR	mismatch repair
NHEJ	non-homologous end joining
PALB2	partner and localizer of BRCA2
SNP	single nucleotide polymorphism
TAI	telomere allelic imbalance

## References

[1] Chatterjee N., Walker G.C. (2017). Mechanisms of DNA damage, repair, and mutagenesis. Environ. Mol. Mutagen..

[2] Alhmoud J.F., Woolley J.F., Al Moustafa A.E., Malki M.I. (2020). DNA Damage/Repair Management in Cancers. Cancers.

[3] Wilczyński J.R., Wilczyński M., Paradowska E. (2022). Cancer Stem Cells in Ovarian Cancer-A Source of Tumor Success and a Challenging Target for Novel Therapies. Int. J. Mol. Sci..

[4] Tung N.M., Robson M.E., Ventz S., Santa-Maria C.A., Nanda R., Marcom P.K., Shah P.D., Ballinger T.J., Yang E.S., Vinayak S. (2020). TBCRC 048: Phase II Study of Olaparib for Metastatic Breast Cancer and Mutations in Homologous Recombination-Related Genes. J. Clin. Oncol..

[5] Koshiyama M., Matsumura N., Konishi I. (2014). Recent concepts of ovarian carcinogenesis: Type I and type II. Biomed. Res. Int..

[6] Mateo J., Lord C.J., Serra V., Tutt A., Balmaña J., Castroviejo-Bermejo M., Cruz C., Oaknin A., Kaye S.B., de Bono J.S. (2019). A Decade of Clinical Development of PARP Inhibitors in Perspective. Ann. Oncol..

[7] Konstantinopoulos P.A., Ceccaldi R., Shapiro G.I., D’Andrea A.D. (2015). Homologous recombination deficiency: Exploiting the fundamental vulnerability of ovarian cancer. Cancer Discov..

[8] Janysek D.C., Kim J., Duijf P.H.G., Dray E. (2021). Clinical use and mechanisms of resistance for PARP inhibitors in homologous recombination-deficient cancers. Transl. Oncol..

[9] McMullen M., Karakasis K., Madariaga A., Oza A.M. (2020). Overcoming Platinum and PARP-Inhibitor Resistance in Ovarian Cancer. Cancers.

[10] Vaidyanathan A., Sawers L., Gannon A.L., Chakravarty P., Scott A.L., Bray S.E., Ferguson M.J., Smith G. (2016). ABCB1 (MDR1) induction defines a common resistance mechanism in paclitaxel- and olaparib-resistant ovarian cancer cells. Br. J. Cancer.

[11] Yap T.A., Plummer R., Azad N.S., Helleday T. (2019). The DNA Damaging Revolution: PARP Inhibitors and Beyond. Am. Soc. Clin. Oncol. Educ. Book.

[12] Ray-Coquard I., Pautier P., Pignata S., Perol D., Gonzalez-Martin A., Berger R., Fujiwara K., Vergote I., Colombo N., Maenpaa J. (2019). Olaparib plus Bevacizumab as First-Line Maintenance in Ovarian Cancer. N. Engl. J. Med..

[13] Kondrashova O., Topp M., Nesic K., Lieschke E., Ho G.-Y., Harrell M.I., Zapparoli G.V., Hadley A., Holian R., Boehm E. (2018). Methylation of all BRCA1 copies predicts response to the PARP inhibitor rucaparib in ovarian carcinoma. Nat. Commun..

[14] Wagener-Ryczek S., Merkelbach-Bruse S., Siemanowski J. (2021). Biomarkers for Homologous Recombination Deficiency in Cancer. J. Pers. Med..

[15] Reid B.M., Fridley B.L. (2021). DNA Methylation in Ovarian Cancer Susceptibility. Cancers.

[16] Paulet L., Trecourt A., Leary A., Peron J., Descotes F., Devouassoux-Shisheboran M., Leroy K., You B., Lopez J. (2022). Cracking the homologous recombination deficiency code: How to identify responders to PARP inhibitors. Eur. J. Cancer.

[17] Rondinelli B., Gogola E., Yücel H., Duarte A.A., van de Ven M., van der Sluijs R., Konstantinopoulos P.A., Jonkers J., Ceccaldi R., Rottenberg S. (2017). EZH2 promotes degradation of stalled replication forks by recruiting MUS81 through histone H3 trimethylation. Nat. Cell Biol..

[18] Miller R.E., Leary A., Scott C.L., Serra V., Lord C.J., Bowtell D., DChang K., Garsed D.W., Jonkers J., Ledermann J.A. (2020). ESMO recommendations on predictive biomarker testing for homologous recombination deficiency and PARP inhibitor benefit in ovarian cancer. Ann. Oncol..

[19] Plon S.E., Eccles D.M., Easton D., Foulkes W.D., Genuardi M., Greenblatt M.S., Hogervorst F.B., Hoogerbrugge N., Spurdle A.B., Tavtigian S.V. (2008). Sequence variant classification and reporting: Recommendations for improving the interpretation of cancer susceptibility genetic test results. Hum. Mutat..

[20] Paik J. (2021). Olaparib: A Review as First-Line Maintenance Therapy in Advanced Ovarian Cancer. Target Oncol..

[21] AstraZeneca Pharmaceuticals LP (2021). LYNPARZA^®^ (Olaparib)—Prescribing Information.

[22] Jackson S.P., Bartek J. (2009). The DNA-damage response in human biology and disease. Nature.

[23] Cooper G.M. (2000). DNA Repair. The Cell: A Molecular Approach.

[24] Rodgers K., McVey M. (2016). Error-Prone Repair of DNA Double-Strand Breaks. J. Cell. Physiol..

[25] Gee M.E., Faraahi Z., McCormick A., Edmondson R.J. (2018). DNA damage repair in ovarian cancer: Unlocking the heterogeneity. J. Ovarian Res..

[26] Hosoya N., Miyagawa K. (2014). Targeting DNA damage response in cancer therapy. Cancer Sci..

[27] Angeli D., Salvi S., Tedaldi G. (2020). Genetic Predisposition to Breast and Ovarian Cancers: How Many and Which Genes to Test?. Int. J. Mol. Sci..

[28] Tomasova K., Cumova A., Seborova K., Horák J., Koucka K., Vodickova L., Vaclavikova R., Vodicka P. (2020). DNA Repair and Ovarian Carcinogenesis: Impact on Risk, Prognosis and Therapy Outcome. Cancers.

[29] Pacheco-Barcia V., Muñoz A., Castro E., Ballesteros A.I., Marquina G., González-Díaz I., Colomer R., Romero-Laorden N. (2022). The Homologous Recombination Deficiency Scar in Advanced Cancer: Agnostic Targeting of Damaged DNA Repair. Cancers.

[30] Mangogna A., Munari G., Pepe F., Maffii E., Giampaolino P., Ricci G., Fassan M., Malapelle U., Biffi S. (2023). Homologous Recombination Deficiency in Ovarian Cancer: From the Biological Rationale to Current Diagnostic Approaches. J. Pers. Med..

[31] Hoppe M.M., Sundar R., Tan D.S.P., Jeyasekharan A.D. (2018). Biomarkers for Homologous Recombination Deficiency in Cancer. J. Natl. Cancer. Inst..

[32] Vergote I., Gonzalez-Martin A., Ray-Coquard I., Harter P., Colombo N., Pujol P., Lorusso D., Mirza M.R., Brasiuniene B., Madry R. (2022). European experts consensus: BRCA/homologous recombination deficiency testing in first-line ovarian cancer. Ann. Oncol..

[33] Hauke J., Horvath J., Groß E., Gehrig A., Honisch E., Hackmann K., Schmidt G., Arnold N., Faust U., Sutter C. (2018). Gene panel Testing of 5589 BRCA1/2-Negative Index Patients with Breast Cancer in a Routine Diagnostic Setting: Results of the German Consortium for Hereditary Breast and Ovarian Cancer. Cancer Med..

[34] Tao M., Wu X. (2021). The role of patient-derived ovarian cancer organoids in the study of PARP inhibitors sensitivity and resistance: From genomic analysis to functional testing. J. Exp. Clin. Cancer Res..

[35] Kondrashova O., Nguyen M., Shield-Artin K., Tinker A.V., Teng N.N.H., Harrell M.I., Kuiper M.J., Ho G.Y., Barker H., Jasin M. (2017). Secondary somatic mutations restoring RAD51C and RAD51D associated with acquired resistance to the PARP inhibitor rucaparib in high-grade ovarian carcinoma. Cancer Discov..

[36] Herzog T.J., Vergote I., Gomella L.G., Milenkova T., French T., Tonikian R., Poehlein C., Hussain M. (2023). Testing for homologous recombination repair or homologous recombination deficiency for poly (ADP-ribose) polymerase inhibitors: A current perspective. Eur. J. Cancer.

[37] Creeden J.F., Nanavaty N.S., Einloth K.R., Gillman C.E., Stanbery L., Hamouda D.M., Dworkin L., Nemunaitis J. (2021). Homologous recombination proficiency in ovarian and breast cancer patients. BMC Cancer.

[38] Gao Q., Zhu J., Zhao W., Huang Y., An R., Zheng H., Qu P., Wang L., Zhou Q., Wang D. (2022). Olaparib Maintenance Monotherapy in Asian Patients with Platinum-Sensitive Relapsed Ovarian Cancer: Phase III Trial (L-MOCA). Clin. Cancer Res..

[39] Pujade-Lauraine E., Brown J., Barnicle A., Rowe P., Lao-Sirieix P., Criscione S., du Bois A., Lorusso D., Romero I., Petru E. (2021). Homologous recombination repair mutation gene panels (excluding BRCA) are not predictive of maintenance olaparib plus bevacizumab efficacy in the first-line PAOLA1/ENGOT-ov25 trial. Gynecol. Oncol..

[40] Goel N., Foxall M.E., Scalise C.B., Wall J.A., Arend R.C. (2021). Strategies in Overcoming Homologous Recombination Proficiency and PARP Inhibitor Resistance. Mol. Cancer Ther..

[41] Onstad M., Coleman R.L., Westin S.N. (2020). Movement of Poly-ADP Ribose (PARP) Inhibition into Frontline Treatment of Ovarian Cancer. Drugs.

[42] Litton J.K., Rugo H.S., Ettl J., Hurvitz S.A., Gonçalves A., Lee K.-H., Fehrenbacher L., Yerushalmi R., Mina L.A., Martin M. (2018). Talazoparib in patients with advanced breast cancer and a germline BRCA mutation. N. Engl. J. Med..

[43] Myriad MyChoice® CDx—Technical Information, Myriad Genetic Laboratories, Inc. https://bit.ly/myChoiceCDxSpecs.

[44] Patel J.N., Braicu I., Timms K.M., Solimeno C., Tshiaba P., Reid J., Lanchbury J.S., Darb-Esfahani S., Ganapathi M.K., Sehouli J. (2018). Characterisation of homologous recombination deficiency in paired primary and recurrent high-grade serous ovarian cancer. Br. J. Cancer.

[45] Palacios J., de la Hoya M., Bellosillo B., de Juan I., Matías-Guiu X., Lázaro C., Palanca S., Osorio A., Rojo F., Rosa-Rosa J. (2020). Mutational screening of BRCA1/2 genes as a predictive factor for therapeutic response in epithelial ovarian cancer: A consensus guide from the Spanish Society of Pathology (SEAP-IAP) and the Spanish Society of Human Genetics (AEGH). Virchows Arch..

[46] Cline M.S., Liao R.G., Parsons M.T., Paten B., Alquaddoomi F., Antoniou A., Baxter S., Brody L., Cook-Deegan R., Coffin A. (2018). BRCA Challenge: BRCA Exchange as a global resource for variants in BRCA1 and BRCA2. PLoS Genet..

[47] Heitz F., Ataseven B., Staniczok C., Denkert C., Rhiem K., Hahnen E., Heikaus S., Moubarak M., Welz J., Dagres T. (2023). Implementing HRD Testing in Routine Clinical Practice on Patients with Primary High-Grade Advanced Ovarian Cancer. Cancers.

[48] Banerjee S., Gonzalez-Martin A., Harter P., Lorusso D., Moore K.N., Oaknin A., Ray-Coquard I. (2020). First-line PARP inhibitors in ovarian cancer: Summary of an ESMO Open-Cancer Horizons round-table discussion. ESMO Open.

[49] Dougherty B.A., Lai Z., Hodgson D.R., Orr M.C., Hawryluk M., Sun J., Yelensky R., Spencer S.K., Robertson J.D., Ho T.W. (2017). Biological and clinical evidence for somatic mutations in BRCA1 and BRCA2 as predictive markers for olaparib response in high-grade serous ovarian cancers in the maintenance setting. Oncotarget.

[50] Lheureux S., Lai Z., Dougherty B.A., Runswick S., Hodgson D.R., Timms K.M., Lanchbury J.S., Kaye S., Gourley C., Bowtell D. (2017). Long-Term Responders on Olaparib Maintenance in High-Grade Serous Ovarian Cancer: Clinical and Molecular Characterization. Clin. Cancer Res..

[51] Chiang Y.C., Lin P.H., Cheng W.F. (2021). Homologous Recombination Deficiency Assays in Epithelial Ovarian Cancer: Current Status and Future Direction. Front. Oncol..

[52] Ruscito I., Dimitrova D., Vasconcelos I., Gellhaus K., Schwachula T., Bellati F., Zeillinger R., Benedetti-Panici P., Vergote I., Mahner S. (2014). BRCA1 gene promoter methylation status in high-grade serous ovarian cancer patients—A study of the tumour Bank ovarian cancer (T.O.C.) and ovarian cancer diagnosis consortium (OVCAD). Eur. J. Cancer.

[53] Swisher E.M., Kwan T.T., Oza A.M., Tinker A.V., Ray-Coquard I., Oaknin A., Coleman R.L., Aghajanian C., Konecny G.E., O’malley D.M. (2021). Molecular and clinical determinants of response and resistance to rucaparib for recurrent ovarian cancer treatment in ARIEL2 (Parts 1 and 2). Nat. Commun..

[54] Callens C., Vaur D., Soubeyran I., Rouleau E., Just P.A., Guillerm E., Golmard L., Goardon N., Sevenet N., Cabaret O. (2021). Concordance between Tumor and Germline BRCA Status in High-Grade Ovarian Carcinoma Patients in the Phase III PAOLA-1/ENGOT-ov25 Trial. J. Natl. Cancer Inst..

[55] Zakrzewski F., Gieldon L., Rump A., Seifert M., Grützmann K., Krüger A., Loos S., Zeugner S., Hackmann K., Porrmann J. (2019). Targeted capture-based NGS is superior to multiplex PCR-based NGS for hereditary BRCA1 and BRCA2 gene analysis in FFPE tumor samples. BMC Cancer.

[56] McDonough S.J., Bhagwate A., Sun Z., Wang C., Zschunke M., Gorman J.A., Kopp K.J., Cunningham J.M. (2019). Use of FFPE-derived DNA in next generation sequencing: DNA extraction methods. PLoS ONE.

[57] Ngoi N.Y.L., Tan D.S.P. (2021). The role of homologous recombination deficiency testing in ovarian cancer and its clinical implications: Do we need it?. ESMO Open.

[58] Pham M.M., Hinchcliff E., Avila M., Westin S.N. (2021). The Clinical Challenges, Trials, and Errors of Combatting Poly(ADP-Ribose) Polymerase Inhibitors Resistance. Cancer J..

[59] Damia G., Broggini M. (2019). Platinum Resistance in Ovarian Cancer: Role of DNA Repair. Cancers.

[60] Pettitt S.J., Krastev D.B., Brandsma I., Dréan A., Song F., Aleksandrov R., Harrell M.I., Menon M., Brough R., Campbell J. (2018). Genome-wide and high-density CRISPR-Cas9 screens identify point mutations in PARP1 causing PARP inhibitor resistance. Nat. Commun..

